# Dextran Formulations as Effective Delivery Systems of Therapeutic Agents

**DOI:** 10.3390/molecules28031086

**Published:** 2023-01-21

**Authors:** Anca Roxana Petrovici, Mariana Pinteala, Natalia Simionescu

**Affiliations:** 1 Centre of Advanced Research in Bionanoconjugates and Biopolymers, “Petru Poni” Institute of Macromolecular Chemistry, 41A Grigore Ghica Voda Alley, 700487 Iasi, Romania; 2 The Research Institute of the University of Bucharest (ICUB), 90 Sos. Panduri, 050663 Bucharest, Romania

**Keywords:** dextran, drug-delivery systems, bioactive compounds, therapeutic effects, biomedical applications

## Abstract

Dextran is by far one of the most interesting non-toxic, bio-compatible macromolecules, an exopolysaccharide biosynthesized by lactic acid bacteria. It has been extensively used as a major component in many types of drug-delivery systems (DDS), which can be submitted to the next in-vivo testing stages, and may be proposed for clinical trials or pharmaceutical use approval. An important aspect to consider in order to maintain high DDS’ biocompatibility is the use of dextran obtained by fermentation processes and with a minimum chemical modification degree. By performing chemical modifications, artefacts can appear in the dextran spatial structure that can lead to decreased biocompatibility or even cytotoxicity. The present review aims to systematize DDS depending on the dextran type used and the biologically active compounds transported, in order to obtain desired therapeutic effects. So far, pure dextran and modified dextran such as acetalated, oxidised, carboxymethyl, diethylaminoethyl-dextran and dextran sulphate sodium, were used to develop several DDSs: microspheres, microparticles, nanoparticles, nanodroplets, liposomes, micelles and nanomicelles, hydrogels, films, nanowires, bio-conjugates, medical adhesives and others. The DDS are critically presented by structures, biocompatibility, drugs loaded and therapeutic points of view in order to highlight future therapeutic perspectives.

## 1. Introduction

Over the last decades, a huge number of macromolecules, including natural polymers, were considered as constituents for drug-delivery systems (DDS) in different formulations: microspheres [1,2], microparticles [3,4], nanoparticles (NPs) [5], nanodroplets [6], liposomes [7], micelles [8,9] and nanomicelles [9], hydrogels [10,11,12], films [13,14], nanowires [15], bio-conjugates [16], medical adhesives [17] and others [18,19,20]. Among natural polymers, polysaccharides are one of the most utilised bio-polymers in DDS’s manufacturing. These compounds are used due to their safety and biocompatibility, the presence of a high variety of chemical functional groups, as well as their high stability and hydrophilic structure. To date, there are a very large number of polysaccharide types isolated and characterised, including dextran (DEX) and its derivatives [3,16], starch and its derivatives [21,22], cellulose and its derivatives [23,24], marine polysaccharides [23], which are used as components in DDS development.

DEX is a noteworthy example of the abovementioned compounds, being a non-toxic, biocompatible, biodegradable and very hydrophilic bio-polymer [25,26]. DEX is biosynthesised intra- or extracellularly by lactic acid bacteria (LAB), which represent one of the most important microbial groups due to their roles in food fermentations and synthesis of techno-functional metabolites [27]. By virtue of its properties, DEX has been used for over 50 years as a circulatory volume expander, in order to improve blood flow [13] and prevent postoperative deep-vein thrombosis [16]. It has also been used in anaemia treatment or as an antiviral agent, being selective for various viruses [13].

In the human body, DEX is degraded by dextranase (1,6-α-D-Glucan 6-glucanohydrolase, E.C. 3.2.1.11) in the liver, spleen, kidney and colon [28,29]. Dextranase endohydrolyses the α-D-(1→6)-glucosidic bonds in DEX resulting in oligosaccharides. The enzyme is synthesised by bacteria present in the colon and after DEX degradation, the by-products are excreted by the kidneys according to the fragments’ molecular weights [30].

In food industries, DEX has technological functions, such as improving the physicochemical properties of food products, and also functional roles, such as prebiotic and immune-modulatory agents [27]. DEX acts as a hydrocolloid in the manufacturing processes of bread and other bakery products, serving as a natural component to replace chemically synthesised commercial hydrocolloids, meeting consumers’ demands for fewer or zero additives in food products. At the same time, it has supplementary properties such as improving dough rheology, textural properties [31] and staling rate [32]. More recently, it was used as a thickener [33], as a surfactant emulsion’s stabiliser [34] and in the production of cereal-based fermented functional beverages and ice cream [35]. The principal potential uses of DEX in foods are mostly related to its capacity to prevent crystallization and retain moisture [36].

In the non-food industry, DEX is used as a bio-separation agent (Sephadex^®^ gels), or as a chromatographic media due to its non-ionic character and good stability under normal operating conditions or for the construction of universal calibration curves used in the evaluation of size exclusion chromatography results [37]. It is used as a steric dispersion stabiliser in the production process of polypyrrole NPs [38].

In the pharmaceutical industry, DEX is already commercially used as a plasma substitute (by increasing volume), as an iron carrier (in the treatment of anaemia, complexed with ferric hydroxide), as an anticoagulant and antithrombotic agent (reducing blood viscosity), as a coating and protective agent for NPs used in nanodrug delivery [25], as an antioxidant and free radical scavenging agent [39], or as inducing agent for interferon biosynthesis [31,35,36,40].

From a medical point of view, the interest in the development and validation of new DDS for different pathologies has grown exponentially. These systems must allow temporal and spatial control of drug delivery, and a continuous plasmatic concentration for a prolonged period and should also improve the drugs’ pharmacokinetic and biopharmaceutical properties. Another very important feature of these systems is that they must provide and increase the drug circulation time and stability in blood flow, improving the drug’s performance, which can be achieved through different types of conjugations with drugs [28].

Over the last decades, DEX has been considered the most promising candidate for the transport of a wide range of therapeutic agents, due to its outstanding physico-chemical properties and biocompatibility [28,41]. Due to the inherent mechanisms of cells which reduce the drug’s effects and facilitate excretion, by using DEX in different DDS, the stability, the local drug concentration and retention time of such nanocarriers (NC) are increased [42].

After systemic administration, the pharmacokinetics of DEX-DDS is considerably influenced by the kinetics of the DEX carrier [41]. Thus, the unmodified polymer can be absorbed by the digestive tract after oral administration only in a small amount. The in vivo studies have shown that both distribution and elimination of DEX depend on the molecular mass and overall charge of the polymer. Pharmacodynamically, the DEX-DDS have resulted in a prolonged effect, a low toxicity profile and a decreased immunogenicity of bioactive molecules [16,43,44]. 

This review presents a critical and comprehensive overview of the recent developments regarding dextran and its applications for the transport and delivery of drugs, proteins, enzymes, imaging agents, nucleic acids, highlighting the substantial increase in therapeutic potential as compared to the free active principles.

## 2. DEX Obtained by Biosynthesis from LAB Fermentation

DEX is a polysaccharide which is biosynthesized intra- or extra-cellularly (endopolysaccharide—ENS or exopolysaccharide—EPS) by several microorganisms such as *Leuconostoc mesenteroides* [31], *Leuconostoc dextranicum* [45], *Lactobacillus brevis*, *Streptococcus mutants* and *Weissella confusa* [33,35,46], *Acetobacter capsulatus*, renamed *Gluconobacter oxydans* and *Acetobacter viscous*, yeasts and moulds (e.g., *Rhizopus* spp.) [36]. Commercially, DEX is usually obtained from *L. mesenteroides* or *L. dextranicum* fermentation in a media with sucrose and a considerable nitrogen source.

In the biosynthesis of linear polysaccharides, there are two general mechanisms. In the first mechanism, the monomers are sequentially added at the non-reducing end of a growing chain using a high-energy donor. This pathway has been demonstrated for DEX biosynthesized by *L. mesenteroides* NRRL-B512F [47]. The second mechanism consists of the sequential addition of monomeric units to the reducing end by insertion between a carrier and the growing chain. In both mechanisms, the DEX molecule grows by extrusion, with the enzyme inserting glucose units from sucrose at one end of the polymer chain [36].

The DEX term describes a large class of bacterial extracellular hydrocolloid homo-polysaccharides [37]. DEX is a complex glycan which can be categorised into three types. The first category is represented by DEX with a main chain of consecutive α-D-(1→6)-linked glucose residues with branching at α-D-(1→2), α-D-(1→3), α-D-(1→4). The second DEX type contains non-consecutive α-D-(1→3) and α-D-(1→6) linear linkages and α-D-(1→3) branch linkages, while the third type contains consecutive α-D-(1→6) linear linkages with α-D-(1→6) branch linkages. The configuration of the DEX molecule influences the biopolymer’s water solubility: polymers with predominantly α-D-(1→6) linkages are the most soluble, while DEX with 43% α-D-(1→3) branch linkages are water insoluble. Moreover, DEX is stable in water, dimethyl sulfoxide, formamide, glycerol, 4-methyl morpholine oxide and hexamethyl phosphamide [36]. 

An important aspect of obtaining high amounts of bio-polymers is the fermentation conditions. Depending on the composition of the culture medium and the strain type, DEX can be obtained with a low or high molecular weight (over 150 kDa) [35,46]. Dextransucrase (1,6-α-D-glucan 6-α-glucosyltransferase, E.C. 2.4.1.5) is a generic name for a family of enzymes that synthesize DEX from sucrose [48]. The activity of dextransucrase is higher in aerobic compared to anaerobic conditions, and the biosynthesis rate are considerably improved by air-sparging [49]. Under proper aeration conditions, sucrose is converted to DEX with maximum yield. Dextransucrase has maximum stability and activity at a pH between 5.0 and 5.5, although most of the published research reports a fermentation pH of around 6.7. At pH 5.5, sucrose is converted into DEX from the beginning of the fermentation process, increasing the conversion yield by approximately 10% in a short period of time [49], preferably in the presence of small amounts of calcium [32]. The optimal biosynthesis temperature range is between 30–45 °C. The enzyme’s nature influences the branching degree of DEX, resulting in different structures of the macromolecule [37]. The molecular weight of biosynthesized DEX is inversely correlated with the dextransucrase concentration and directly correlated with sucrose concentration and temperature [50]. Actually, the dextransucrase cleaves the glycoside bond in sucrose, releasing glucose which is further used in the biosynthesis of DEX by natural polymerisation, and fructose which is used as an energy source in different metabolic processes [51].

To increase the EPS biosynthesized amount, research groups generally optimise the culture media composition by supplementing it with additional carbon and nitrogen sources [52]. Han et al. (2014) [31] obtained 32 g/L DEX from *L. mesenteroides* BD1710 fermentation in culture media containing tomato juice supplemented with 15% sucrose. Another considerable amount of DEX, about 25.2 g/L, was obtained in our laboratory by *W. confusa* PP29 fermentation in culture media containing UHT milk supplemented with 8% sucrose [35]. This compound had a remarkable disrupting effect on the biofilm produced by *Candida albicans* SC5314 strain, as well as no cytotoxic effect on normal human dermal fibroblasts (NHDF) [35]. Wang et al. (2022) [53] simultaneously obtained DEX and vitamin B12 by using *Propionibacterium freudenreichii* DSM 20,271 and *Weissella confusa* A1 in a soya flour- or rice bran-based media supplemented with sucrose. The aim of the study was to obtain bread with high nutritional value and the results also showed that the obtained DEX amount was very high, at approximately 58 g/L [53]. Experiments performed in our laboratory showed that the addition of aqueous fruit extract from *Hippophae rhamnoides* to the LAB culture media yielded 4.8 g/L dry EPS, with 2 g/L more compared with standard MRS media [54], while the addition of anthocyanin-rich *Hibiscus sabdariffa* L. extracts to culture media supplemented with peptone and sucrose yielded biosynthesized DEX with high molecular weights [55] (see Table 1). 

## 3. Biomedical Applications of Modified DEX

After thorough investigations, different research groups postulated that pure DEX-based systems cannot achieve good mechanical properties and high drug-loading capacity. Native DEX exhibits low-cell-adhesive properties and in order to obtain hydrogels with controlled cell-scaffold interactions, specific molecules must be incorporated [19]. Many research groups have chemically modified DEX by introducing functional groups into the molecule through cross-linking reactions, therefore improving mechanical strength and drug-loading ability [9,41] and increasing the number of compound classes that can be obtained. Furthermore, DEX has been shown to have metal chelating activity [46] and antioxidant properties [59], as well as antitumour activity by regulating apoptosis and autophagy [61].

Below we present the most commonly used types of modified DEX, as well as the active substances that have been loaded into DEX-based systems.

### 3.1. Acetalated Dextran (Ac-DEX)

The main reason for performing DEX acetylation is to allow solubility of DEX molecules in organic solvents, facilitating the encapsulation of various hydrophilic and hydrophobic active substances, which has always been challenging, and allowing their simultaneous delivery [62]. Ac-DEX is an essential derivative of DEX synthesized in mild conditions, at room temperature, from DEX and 2-methoxypropene in a one-step reaction catalysed by pyridinium p-toluene sulfonate [3]. Ac-DEX contains cyclic and methoxy acyclic acetal moieties and has been shown to be biodegradable at neutral pH, biocompatible and pH-sensitive [4,62]. Because it is an acid-sensitive polymer, Ac-DEX degrades more rapidly at lower pH, for example in the endosome of phagocytic cells, tumours, or in areas with inflammation [63], making it an ideal carrier for a wide range of therapeutics. Ac-DEX has several characteristics that make it a unique biodegradable polymer, such as facile synthesis and degradation rates’ adjustment properties. It is suitable for vaccine applications, targeted host-directed therapies to macrophages, controlled release of drugs, chemotherapeutic delivery and engineered drug-delivery devices [64]. By the simultaneous release of different active substances, synergistic effects, as well as the reduction in side effects and solubility improvement could be achieved at lower concentrations and improved pharmacokinetics [62]. 

As a therapeutic system, Ac-DEX was used to develop porous microparticles made by single emulsion method in water/oil and loaded with rapamycin [4,65], camptothecin [66], or curcumin [67] in order to be used for pulmonary drug delivery or phagocytes’ passive targeting. The delivery and release tests recorded very good results. These systems are more efficient in drugs’ transport to the alveolar region of the lung, or for immune suppression therapies than other similar systems [4,65,66,67]. At the pulmonary level, after the post-processing of these microparticles, the respirable fraction increased with the improvement of aerosolization and no significant damage was caused by the system to lung epithelial cells either in liquid- or air-exposed conditions [4,65,66,67]. The dry powder aerosol formulations were capable of deep lung delivery of drugs by targeting and releasing the therapeutics to a desired location [4,65,66,67]. By using these systems, a rapid onset of pharmaceutical action was obtained, avoiding hepatic metabolism and decreasing the side effects of the drugs. Resiquimod, a drug with antiviral and antitumour activity, was encapsulated in an electrospun Ac-DEX microparticles’ scaffold and the results were remarkable for tissue engineering, wound healing, immunotherapy and drug-delivery applications [68,69]. Pyraclostrobin, an antifungal agent, was successfully loaded in pH-sensitive Ac-DEX microparticles in order to treat *Sclerotinia sclerotiorum* plant infections [3]. Konhäuser et al. (2022) [62] developed a DDS system in order to simultaneously release L-asparaginase and etoposide. The active substances have synergistic activity against chronic myeloid leukaemia (CML) K562 cells, but L-asparaginase is hydrophilic and etoposide is hydrophobic [62]. This system has great potential for CML therapy due to its ingenious ability to release both compounds in a pH-dependent manner, leading to synergistic cytotoxicity, increased drug efficacy and reduced side effects [62].

### 3.2. Oxidized Dextran (oDEX)

Some research groups have obtained oDEX in order to bind therapeutic active molecules for secure delivery. DEX oxidation using sodium periodate is a catalysis-free aqueous reaction which produces a polyaldehydic DEX that can serve as a macromolecular cross-linker for amino groups-bearing substances.

By using oDEX, different DDS were synthesized, including microspheres, vesicles, hydrogels, NPs. Cortesi et al. (1999) [1] synthesized oDEX gelatine microspheres loaded with TAPP-Br antitumour drug and cromoglycate, obtaining very good results for drug release. Curcio et al. (2020) [70] developed a self-assembling oDEX-based vesicular system loaded with camptothecin, which was determined to be very efficient against MCF-7 and MCF-10A cell lines. The antitumour drugs, such as 5-fluorouracil and methotrexate, were encapsulated in oDEX hydrogels for breast, skin and gastrointestinal tract cancer treatment [71]. The obtained DDS induced faster drug release and had excellent biocompatibility and degradability, therefore being suitable for anticancer therapies [71]. Novel oDEX-based NPs for insulin release [29] or loaded with 5-fluorouracil for colorectal cancer therapies [30] were also obtained and were suitable for further in vivo testing.

Zhou et al. (2022) [12] reported an oDEX-based hydrogel loaded with black phosphorus nanosheets and zinc oxide nanoparticles. This DDS was suggested to be a hopeful approach for chronic wound treatment with bacterial infection through the synergistic effect of photothermal action and immunomodulation [12]. Multiple hydrogels as transdermal DDS loaded with ceftazidime or with collagen and Epidermal Growth Factor were reported for the treatment and healing of diabetic wounds infected with multidrug-resistant bacteria [39,72].

### 3.3. Carboxymethyl Dextran (CMD)

CMD, a polyanionic polysaccharide, was considered as a DDS constituent since it was discovered that its functional groups facilitate chemical conjugation and ionic complexation with various drugs. Its hydrophilic characteristics facilitate prolonged drug circulation improving its tumour-targeting efficiency [73]. By itself, CMD has high antioxidant properties [74].

CMD was used as a nanocomposite hydrophilic shell in order to be loaded with glutathione as an inhibitor of reactive oxygen species’ cytotoxic effects associated with tumour apoptosis [75].

Magnetic NPs were coated with CMD in order to be used as contrast agents for magnetic resonance molecular imaging (MRI) [76,77]. Several research groups used CMD-coated magnetic NPs loaded with antibodies [78], peptides [79] and enzymes [80] for different medical applications.

### 3.4. Dextran Sulphate Sodium (DSS)

Certain types of dextran functionalization can lead to very toxic compounds, which can, however, be useful for particular applications. DSS is a polyanionic derivative of dextran with high-water solubility properties containing approximately 17% sulphur with up to three sulphate groups (-OSO_3_Na) per glucose molecule [81]. DSS has found wide utilization in the food, biotechnology, cosmetic and pharmaceutical industries [82]. In proper concentrations, it exhibits positive effects as an anticoagulant and antiviral agent or has the properties of lowering blood lipid and glucose levels in clinical studies [83]. Despite DSS promising application prospects and biological properties, its application is limited due to its harmful effects on the gastrointestinal tract [83]. 

Different research groups use DSS to induce colitis, thus creating artificial conditions for studying inflammatory bowel diseases, such as ulcerative colitis and Crohn’s disease. The colitogenic potential of DSS depends on its molecular weight which must be between 36–50 kDa. DSS produces manifestations associated with inflammatory bowel disease, such as submucosal erosions, ulceration, inflammatory cell infiltration, crypt abscesses, as well as epithelioglandular hyperplasia [81]. It also determines the shrinkage of colon length and increases the relative colon weight/length ratio accompanied by mucosal oedema and bloody stools [81]. The DSS colitis paradigm is the most appropriate model for the human phenotype, from many points of view. For this injury, many drugs were tested as treatment, including curcumin [84], garlic oil (which has antioxidant, anti-inflammatory and immunomodulatory effects) [85], carvacrol (a phenolic monoterpene extracted from *Oreganum vulgarea* sp. essential oils with antioxidant, anti-inflammatory and anticancer properties) [86], resveratrol [87], glucose-lysine Maillard reaction products [88], liquorice (a *Glycyrrhiza uralensis* rhizome-derived product with anti-inflammatory activity) [89], *Lactobacillus sakei* K040706 (with immuno-stimulatory effects) [90] and *Polygonum tinctorium* leaves extract (by enhancing the mRNA expression of interleukin-10 and decreasing expression of tumour necrosis factor in colon tissues) [91]. 

DSS has also been used for film coatings with biological and biomedical applications [13]. Mixed DSS-based systems were developed, such as eco-friendly PVA/DSS nanofibers loaded with ciprofloxacin [18] or chitosan-DSS microparticles loaded with a hydrophilic peptide used as immunity-enhancing adjuvant or considered as vaccine electuary [92].

An antibacterial biocapsule system obtained from multilayer self-assembled diethylaminoethyl (DEAE)-DEX hydrochloride and DSS was developed as a DDS for kanamycin-resistant *Escherichia coli* treatment. The system manifested an inhibitory effect during bacterial growth having high potential as an antimicrobial agent in future treatments against infection [20].

Wang et al. (2020) [93] developed a dual DDS for paclitaxel and 5-fluorouracil. The pH-sensitive system exhibited a controlled release profile based on a mechanism following a two-phase kinetic model [93]. The system’s efficiency was investigated on HepG2 cells, resulting in synergistic effects between the two drugs and enhanced inhibition of cancer cells, presenting a good potential for biomedical delivery applications [93].

### 3.5. Diethylaminoethyl-Dextran (DEAE-DEX)

DEAE-DEX was the very first chemical vector used for DNA delivery, reported by Vaheri and Pagano in 1965 as DEAE-DEX used to enhance the cells’ viral infectivity. The DEAE-DEX-mediated transfection method gained attention in the early 1980s because of the simplicity, efficiency and reproducibility of the procedure. DEAE-DEX forms electrostatic interaction complexes with DNA, exhibiting higher transfection efficiency, but at high concentrations, it is toxic to cells [94]. Recently, it was used to develop carrier polyplex nanoparticles with luciferase coding mRNA [95] or used for β-interferon production enhancement [40].

## 4. Dextran Used in Drug-Delivery Systems

From a structural point of view, as a bio-polymer, DEX has molecular weights higher than 1000 Dalton, and a linear backbone of α-linked D-glucopyranosyl repeating units [28]. DEX contains a large number of hydroxyl groups which are capable of conjugating bioactive molecules by direct coupling or via a linker. DEX has been used to form hydrogels [10,11,12], films [13,96], nanosystems (by itself or as a coating agent) [5,6,9,15,16] and other systems [7,8,17,18,19,20], in order to release controllable amounts of drugs (Table 2). Recently, it was demonstrated that DEX has a protective effect on cells against oxidative stress induced by drug cytotoxicity [28,42]. 

It has been postulated that in vivo drug concentrations need to be as constant as possible and optimally targeted to specific cells or organs in order to avoid prolonged treatments. Microencapsulation of antineoplastic drugs has been done using natural or synthetic polymeric materials with the aim of maintaining constant and high drug levels in the blood or at the tumour site, thus reducing multiple administrations and possibly targeting the active agents to the desired location [1]. 

Below, the most used systems containing DEX as a component have been reviewed.

### 4.1. DEX as a Hydrogel Component

The use of natural polymers in hydrogel systems’ development can confer highly beneficial properties to drugs. By using DEX, optimal release profiles and desirable therapeutic characteristics can be achieved for a wide range of DDS [28]. Hydrogels as polymeric networks with swelling capacity can be biodegradable or not, and drugs can be encapsulated in these structures, obtaining delivery systems with controlled drug release [97]. 

DEX-containing hydrogels are considered valuable and sustainable biomaterials for biomedical applications [10]. They are being used extensively in the pharmaceutical and biomedical fields for drug delivery, tissue engineering [10], neovascularization [106], regenerative medicine, wound repair and dressings [12,41,107], due to DEX’s lubrification and unique soft-wet properties similar to natural extracellular matrices [108], as well as their advantages for commercial production, such as high yields and low costs [35] (Table 2).

Traditional antibacterial hydrogels deliver large dosages of antibiotics or other drugs, increasing the risk for cytotoxicity. However, some research groups have used antimicrobial agents with synergistic activity in models of normal and diabetic wounds infected with multidrug-resistant bacteria, achieving higher therapeutic effects at lower doses compared to classical antibiotics [72].

### 4.2. Dextran as NP Component or Coating Agent

Over the years, intensive efforts have been made to design intelligent systems that are able to deliver drugs more efficiently to the target site and at the same time to minimise the side effects. NPs as DDS for enhancing the drugs’ therapeutic efficiency are the hot spot of research in the field of nano-biotechnology. Although there are many advantages associated with these NPs, such as increased solubility of hydrophobic drugs favouring long circulation times in the blood or higher bioavailability [109,110], there are still a number of drawbacks, such as burst release, limited stability of formulations leading to drug leakage and nonspecific cellular uptake resulting in undesired adverse effects [9,44]. Most NPs can be tailored for specific site targeting, controlled release of drugs and high stability under different administration routes. NPs have the ability to penetrate easily through fine blood capillaries due to their subcellular and nano sizes [29,111]. Furthermore, drugs have often been covalently bonded to natural or synthetic polymers in order to reduce renal excretion [109].

DEX in its native form does not self-assemble into NPs, but nonetheless has high water retention capacity and heavy metal chelating activity for Zn^2+^, Fe^2+^, Cu^2+^, Cd^2+^ and Pb^2+^ [46]. Different strategies have been developed in order to fabricate DEX-based NPs for drug delivery (Table 3), among which we can mention the covalent functionalization of DEX hydroxyl groups or crosslinking of DEX through the lateral hydroxyl groups (using a variety of crosslinking reactions and linkers), both necessary for physical self-assembly into NPs [112] or reducing in vivo accumulation and clinical risk [30,96,113,114].

In order to safely deliver a drug and to release the correct dose, first of all, it is mandatory to study the physico-chemical properties of the administered drug in the location of interest. Furthermore, in order to selectively target a specific site, it is imperative to investigate the physiological properties of the microenvironment. The toxicity and the bio-distribution of a delivery system are influenced by the chemical nature of the components, system’s size and the coating agents [125]. By using DEX as a coating agent for any NPs, the interactions with cells and proteins are limited, thus conferring increased circulating half-life and colloidal stability in biological environments, which in turn determines good overall safety in vivo and no visible tissular damage [96,129]. At the same time, by the encapsulation of the drug in these systems, the side-effects of the drug are minimized, the efficiency is enhanced and the drug can be released in a controlled rate depending on the drug’s diffusion coefficient [44,71,120,124].

### 4.3. Dextran as Nanocarrier Component

Nanocarriers (NC) are similar to NPs, but the methods of synthesis are different. Thus, reaction components represented by natural polymers with low molecular weights and various molecules with smaller or larger molecular weights are embedded by chemical or physical processes [44,130]. Next, the final synthesised compound self-assembles through hydrogen interactions or electrostatic attractions in a NC system. Natural or synthetic hydrophobic substances with therapeutic activity are encapsulated either in the core or grafted on the NC surface by chemical reactions or by electrostatic interactions [131].

Similar to NPs, NCs also help improve drug efficacy, having the ability to increase drug absorption in tissue and increase cellular uptake, to protect the drug from degradation and interaction with the biological environment and to control the drug’s pharmacokinetic distribution profile [132]. NCs such as liposomes, micelles or polymeric NPs have shown fabulous opportunities in the field of targeted drug delivery for cancer therapy [133]. Table 4 presents DEX-based NCs developed for drug delivery.

### 4.4. Dextran as Micelles’ Component

Micelles are a type of highly regarded DDS, especially for the delivery of hydrophobic/lipophilic drugs due to their unique physicochemical properties, containing a hydrophobic core and a hydrophilic shell. Natural polymeric micelles are more widely used in novel DDS due to their biocompatibility and tunable properties [8]. These DDS have a great capacity to encapsulate high amounts of bioactive compounds and to deliver them at targeted locations in the body.

Several groups have developed DEX-based micelles for drug delivery in a variety of pathologies. Zhang et al. (2020) [137] developed a self-assembled pH-responsible micelle formed by conjugated DEX loaded with doxorubicin and found that the drug accumulation in tumours was increased due to permeation enhancement. Jin et al. (2017) [138] tested the cytotoxicity and antitumour activity of their system on MCF-7 and SKOV-3 tumour cells in vitro and the results were promising. Later, a self-assembled DEX-based micelle was loaded with rapamycin, decreasing the drug’s toxicity and increasing the system’s uptake by tumoral cells, without affecting normal cells’ viability [9]. Malekhosseini et al. (2020) synthesized DEX-based micelles which had a hydrocortisone encapsulation efficiency of 79% and 90% drug release in the first 12 h with cell viability higher than 90% [8]. The study of nateglinide and insulin, vitamin E succinate and insulin combinations loaded into DEX-based micelles reduced oxidative stress and improved the mitochondrial function and glucose metabolism, while also improving the cognitive capacity of mice, demonstrating a paradigm for specific and high-efficacy combination therapy for Alzheimer’s disease [139].

## 5. Conclusions

Dextran is a biosynthesized non-toxic, biocompatible and biodegradable macromolecule which has been extensively used as a major component in many types of DDS due to its versatile properties. Numerous DDS obtained so far using dextran have great potential in different pharmaceutical applications but, in order to maintain the high DDS biocompatibility, the use of dextran obtained by fermentation with minimum chemical modifications is recommended. By performing dextran chemical modifications, artefacts can appear in the DEX spatial structure which can further lead to biocompatibility decreasing or even cytotoxicity increasing. As a result, many DDS containing acetalated, carboxymethyl, diethylaminoethyl-dextran, or dextran sulphate sodium salt have been removed from in vivo or clinical studies.

On the other hand, the multitude of developed DDS (microspheres, microparticles, nanoparticles, nanodroplets, liposomes, micelles, hydrogels, films, nanowires, bio-conjugates, medical adhesives and others) have considerably increased the type and number of applications compatible with DEX-DDS. However, there is still a need for continuous DDS development in order to optimize and study as many systems as possible for biomedical and pharmaceutical applications. 

## Figures and Tables

**Table 1:** Biosynthesized DEX amount and molecular mass depending on culture media composition.

Strain	Culture Media	Fermentation Conditions	Dry DEX Amount, g/L	Molecular Mass, Da	References
*Leuconostoc mesenteroides* ZDRAVLJE SR-P	Sucrose, yeast extract, barley malt extract, Na_2_HPO_4_ • 12 H_2_O, MgSO_4_ • 7 H_2_O, KCI, supplemented with 12% sucrose	200 rpm	54.9		[49]
*Leuconostoc mesenteroides* BD1710	Tomato juice with 15% sucrose	48 h at 28 °C	32.0	6.35 × 10^5^	[31]
*Weissella confusa* PP29	MRS, sucrose (80) dissolved in UHT milk	48 h at 33 °C	25.2	1.2 × 10^6^	[35]
LAB-PP15	MRS, sucrose (80) dissolved in UHT milk	48 h at 33 °C, 100 rpm	9.0	1.9 × 10^5^	[56]
*W. confusa* H2	MRS	48 h at 30 °C		2.705 × 10^6^	[46]
*W. cibaria* SJ14	Modified MRS semi-defined medium	34 h at 37 °C	0.33	7.12 × 10^4^	[57]
*Leu. pseudomesenteroides* DRP-5	MRS agar	36 h at 30 °C		6.23 × 10^6^	[58]
*Leuconostoc mesenteroides* BI-20,	FYP broth with 3% sucrose	48 h at 30 °C		1 × 10^8^	[27]
*Weissella confusa* A16	Soya flour or rice bran with 10% sucrose	24 h at 25 °C, 150 rpm	58.0		[53]
*Lactobacillus kunkeei* AK1	FYP broth with 3% sucrose	48 h at 30 °C		45 × 10^3^	[59]
*Weissella cibaria* NC516.11	Distiller grains of Fenjiu	24–48 h at 37 °C		2.82 × 10^6^	[60]

**Table 2:** Dextran applications in drug-delivery systems.

DDS Type	Drug Loaded	Targeted Disease/Applications	Observations	Reference
Hydrogel	Polydopamine	Multidrug-resistant bacterial infections	Good physical and chemical properties; low cytotoxicity against mouse fibroblast cells; precise in vivo antibacterial and wound-healing performance	[41]
Nanohydrogel matrix	Maghemite		Magnetic properties; high drug loading and stability in the circulatory system	[97]
Hydrogel	Aniline trimer elastomer	Smart DDS for localised drug release	Controllable swelling ratio; stable rheological properties; good conductivity; electric stimuli-dependent activity	[10]
Nanogel	Methotrexate	HeLa cells	Sensitive to the variation of the pH and redox environment; high release rate at pH 5.0; suitable carriers for cancer chemotherapeutics	[98]
Magnetic microgels	Doxorubicin		Promising results for further studies	[99]
Nanogels	Doxorubicin	H1299 cancer cell line	The indisputable results promote this system for further in vivo testing	[100]
Hydrogels	Praziquantel	Anthelmintic disease	Good in vitro results	[28]
Hydrogels	Ondansetron^TM^	Antiemetic following chemotherapy	Good release kinetics’ curve	[101]
Cryogels	Vitamin B12	Vitamin B12 deficiencies	Suitable carriers for water-soluble biomolecules’ delivery	[102]
Micro-hydrogel	Indole; 3-nitrophenol; hydroxybenzoic acid; diclofenac;		Very satisfactory release kinetics’ curve	[2]
Nanohydrogels	Ornidazole	*Clostridium* sp. infections	Very good in vitro antibiotic effect	[103]
Nanogel	Curcumin	New foods development	In vitro simulations showed sustained drug release	[104]
Nanogel		Food ingredient preparation	High potential for hydrophobic bioactive compounds’ encapsulation	[105]
Hydrogels	Arginine-glycine-aspartic acid (RGD) sequences	Artificial cardiac tissues	Promising system for building cardiac grafts	[19]
Hydrogels	RGD and activin A	Ovarian tissue culture	Significantly improves follicular oocytes’ in vitro maturation and development; synergistic effects in 3D tissue culture development	[106]

**Table 3:** DEX-based NPs developed for drug delivery in different pathologies.

DDS Type	Drug Loaded	Targeted Disease/Application	Observations	Reference
NP	Lidocaine		Very good drug-release results	[115]
NP	Model protein and antibodies	Cardiovascular pathologies	A promising tool for further in vivo tests	[116]
Magnetic NP-DEX coated	Protocatechuic acid	Vascular inflammation	Very good in silico results	[117]
Magnetic NP-DEX coated	Protocatechuic acid	Vascular inflammation	Very good in vitro results	[118]
NP	5-fluorouracil	Skin damage	Less immunogenic compared with other systems	[110]
NP	5-fluorouracil	Colorectal cancer	The HCT116 colon cancer cell line treatment was efficient.	[112]
NP	Doxorubicin		pH/redox-responsive, self-assembly in aqueous solutions; excellent plasmatic stability and anti-protein adsorption ability for tumour cellular uptake.	[113]
NP	Dodecilamine and doxorubicin		pH-sensitive drug release	[119]
NP	Doxorubicin		pH-sensitive intracellular drug release in HeLa cells	[120]
NP	Doxorubicin		Acid-responsive NP in water; loaded system toxicity on HeLa cells is comparable to the drug’s;	[63]
NP	Doxorubicin	Human cervix carcinoma cells (HeLa)	No DDS cytotoxicity and structural stability under the simulated physiological conditions; drug release in acidic conditions; very good in vivo results	[121]
NP	Amphotericin B	*Candida albicans* infection	No loaded DDS toxicity compared with free drug. Very good results	[111]
NP	Bovine serum albumin, granulocyte-macrophage colony-stimulating factor (GM-CSF), granulocyte colony-stimulating factor (G-CSF), β-galactosidase and myoglobin	Protein stabilization for pharmaceuticals applications	DEX NPs can preserve the protein’s bioactivity during the preparation process; DEX NPs attenuate the acidic microenvironment by means of the dilution effect;	[122]
NP	Insulin	Diabetes	Very good results	[123]
Magnetic NP-DEX coated	Propiconazole	*Candida albicans* infection	Direct interaction with the cell wall in both planktonic and biofilm phases; 77% biofilm breakdown	[117]
Magnetic NP-DEX coated	Folic acid	Magnetic resonance imaging	Negative contrast agent for antigen allowed arthritis visualisation in a rat model and measuring the treatment response	[114]
NP-DEX coated		Human epithelial colorectal adenocarcinoma cells	Good anticancer effect	[124]
Gold NP-DEX coated		Solid carcinoma and Ehrlich ascites carcinoma transplanted on mice	Significant antitumour effects; Improvement of body functions; increased liver antioxidant properties; increased the B-cell lymphoma 2 gene expression level; suppressed the apoptotic pathway	[125]
NP-DEX coated	Zidovudine	Viral infection	Increased drug half-life; well internalized in the neural cells	[16]
NP	Myristoyl- ECGKRK peptide	Cancer therapies	Satisfactory results obtained	[126]
NP	Chloroquine diphosphate	*Plasmodium falciparum* malaria infection	Very good antimicrobial effects obtained DDS suitable for in vivo tests	[127]
NP	Curcumin	Breast cancer	DDS has good drug-loading and delivery performance; very effective against MCF-7 cell line	[128]

**Table 4:** DEX-based NCs developed for drug delivery.

DDS Type	Drug Loaded	Targeted Disease/Applications	Observations	Reference
NC	Camptothecin	Cancer therapies	High drug-loading rate; superior stability in aqueous solutions; notable in vitro antitumour activity against HeLa and MCF-7 cells	[130]
NC	Choline kinase siRNA	siRNA cancer therapy	Successful delivery of siRNA	[131]
DEX-coated graphene oxide NP	Curcumin	MCF-7 breast cancer cell lines	Very good results obtained; potential DDS for chemotherapy application	[44]
NC	Paclitaxel and silybin	A549 lung cancer cells	Excellent encapsulation efficiency of both active substances; employs synergistic effects through chemotherapy sensitization and microenvironment modulation, improving the efficacy of cancer therapy; in vivo tests confirmed tumour growth inhibition	[25]
Conjugate	Calcium ions	Calcium supplements’ carrier	Could be used as an effective carrier for new calcium supplements	[134]
Nanowires		Pharmaceutical applications	Useful biomaterial for medical applications	[15]
NC	Cabazitaxel	Prostatic cancer	Promising DDS as a substitution for the current market formulation	[135]
Conjugate	Metronidazole	Protozoa infection	Very good in vivo results	[136]

## Data Availability

Not applicable.
